# Film Thickness and Glycerol Concentration Mapping of Falling Films Based on Fluorescence and Near-Infrared Technique

**DOI:** 10.3390/mi13122184

**Published:** 2022-12-09

**Authors:** Isabel Medina, Stephan Scholl, Matthias Rädle

**Affiliations:** 1 Center for Mass Spectrometry and Optical Spectroscopy, Mannheim University of Applied Sciences, Paul-Wittsack-Straße 10, 68163 Mannheim, Germany; 2 Institute for Chemical and Thermal Process Engineering, Technische Universität Braunschweig, 38106 Braunschweig, Germany

**Keywords:** concentration distribution imaging, falling film, film thickness distribution imaging, fluorescence, glycerol, multiwavelength, near-infrared, water

## Abstract

Falling film evaporation processes involve high fluid velocities with continuous variations in local film thickness, fluid composition, and viscosity. This contribution presents a parallel and complementary film thickness and concentration mapping distribution in falling films using a non-invasive fluorescence and near-infrared imaging technique. The experiments were performed with a mixture of glycerol/water with a mass fraction from 0 to 0.65 $g_{glyc}g_{total}{}^{-1}$ and operating ranges similar to evaporation processes. The measurement system was designed by integrating two optical measurement methods for experimental image analysis. The film thickness was evaluated using a VIS camera and high-power LEDs at 470 nm. The local glycerol concentration $g_{glyc}g_{total}{}^{-1}$ was determined using a NIR camera and high-power LEDs at 1050, 1300, 1450 and 1550 nm. A multiwavelength analysis with all NIR wavelengths was implemented with a better correlation for falling films at low flow velocity. The results show an improvement in the analysis of falling films with high flow velocities up to almost 500 mm/s by using only the 1450 nm wavelength and the fluorescence measurement. Simultaneous imaging analysis of film thickness and concentration in falling films provides further insight into understanding mass and heat transport and thus supports the optimization of falling film evaporators.

## 1. Introduction

Heat and mass transfer are essential parts of industrial separation processes and represent a crucial engineering task. The evaporators are the most extensively used single-production equipment in these processes. For this reason, describing these apparatuses and their product behavior is crucial for design and operation to avoid product damage. Gentle partial evaporation of the product medium is decisive for product quality, and falling film evaporators are used for this purpose, especially in industrial processes like food production and the evaporation of sucrose solutions [1,2], milk, and fruit juices [3,4,5,6,7,8]. Other applications include solvent separation from oligomers or polymers [9,10,11], separation of petrochemical naphtha [12], and dewatering of ionic liquids [13,14,15].

In comparison to other evaporator types, falling film evaporators allow for a lower minimum driving temperature difference at very low local liquid hold-up. These two aspects minimize the thermal load on the treated products, enabling gentle evaporation of the product. For this purpose, falling film evaporators operate with a low film thickness between 0.2 and 2 mm. An increase in film thickness in falling films results in reduced performance and thus in an insufficient heat transfer or evaporation rate. Complete wetting of the evaporator surface is also crucial for evaporation. In non-wetted areas, the local temperature would increase, resulting in fouling or crystallization, such as in the evaporation of milk or sugar solutions [16,17,18,19]. Not only tracking the film thickness or waviness is decisive in determining the heat transfer quantity but also the change in substance composition. While the boiling temperature of a pure substance is defined only by the vapor pressure, the boiling temperature of a mixture also depends on the composition of the liquid phase.

The local film thickness has been investigated in many studies to characterize the falling film. Damsohn and Prasser classify the methods into four groups: extraction, photons, ultrasound, and electrical [20]. Dupont later added a fifth group, neutron [21], and his classification is based on the work of Clark [22]. Tibiriçá et al. also presented a summary of measurement techniques for microscale two-phase systems [23].

The extraction methods are based on measuring the initial amount, the mass of the total distillate, or the initial product and comparing these after the process. Nußelt used fluid weighing to propose his water film theory [24,25]. Mechanical methods were implemented by Hopf using fine screws to determine film thickness with a sucrose solution [26].

Electrical methods include techniques such as the needle-contact probe and the conductivity or electrical capacitance measurement to determine film thickness. These methods estimate film thickness and two-dimensional (2D mapping) information [20,27,28,29,30,31].

The chromatic confocal sensing technique is a non-invasive method used by Zhou and T. Gambaryan-Roisman to measure film thickness in wavy falling films for Reynolds numbers between 206 and 317 [32]. Schröder also used this measurement technique to analyze the flow characteristics of an excited falling film with a measurement frequency of up to 4 kHz and a few micrometers of local resolution [33].

Dumin studied the growth of the film thickness of monosilane in a chamber with a quartz window using infrared analysis [34]. Porter et al. presented a mid-infrared laser absorption method for measuring fuel vapor phase and film thickness with a maximum of 30 µm [35].

The fluorescence intensity technique is widely used to measure film thickness in falling films [36,37]. Hewitt et al. studied film flow in a two-phase system with different techniques and the fluorescence method [38,39]. Liu et al. presented a two-dimensional film distribution in the laminar-to-laminar-wavy region [40]. Heavens and Gingell also used the fluorescence technique to measure magnesium fluoride film thicknesses down to 1 nm [41]. Greszik et al. used the laser-induced fluorescence (LIF) method and Raman imaging to measure the film thickness of water between 0 and 0.5 mm [42].

The application of image analysis in chemical engineering processes has increased in recent years. The availability of low-cost camera systems with a high acquisition rate and resolution allows the application in research and industry. In fluorescence and UV/VIS spectroscopy, cameras are often used as sensors for analysis.

Al-Sibai experimentally investigated trickle film flow and heat transfer characteristics using a fluorescence intensity method. Coumarin 152a is used as a fluorescent indicator excited with a laser diode at 405 nm. The fluorescence is emitted with a maximum peak at 460 nm and received by a photomultiplier [43,44,45]. Lel et al. used the fluorescence measurement technique and chromatic confocal image analysis to determine the wave velocity and film thickness of DMS-T01.5, T05, and T12 silicone oils at Reynolds numbers between 2 and 700 [44]. Schagen and Modigell applied a method for parallel film thickness measurement and temperature distribution [46,47]. Lu et al. use the same measurement method to determine the influence of two fluid distributors on the wetting degree and film thickness of falling films. The fluorescein sodium salt is excited with UV illumination, and a camera was used as a sensor [48]. 

Near-infrared spectroscopy and the development of new and inexpensive cameras enable image analysis in the near-infrared range. For this purpose, a near-infrared camera is commonly utilized in conjunction with a light source such as a halogen lamp or light source with different wavelengths, e.g., LEDs. Common solvents, especially water and alcohols, have characteristic absorption bands in the near-infrared spectral range, measured with the camera. For example, water has an individual absorption spectrum with the highest peak between 1440–1460 nm [49]. Therefore, this method is ideal for measuring water film thickness [50,51,52,53]. 

In recent literature, Dupont and Lubnow et al. focused their work on image analysis using the near-infrared measurement technique to determine film thickness [21,54]. Dupont only used one NIR wavelength narrowband filter. Lubnow implemented near-infrared imaging using four wavelengths with a filter wheel, increasing the image acquisition time. Schmidt et al. investigated the dynamic changing of fluid thickness by using an absorption-based laser sensor and the near-infrared spectra of liquid water for film thickness up to 440 µm [55]. Film thickness measurements in evaporation and flow processes of the refrigerant R1233zd by absorption spectroscopy in the near-infrared area were studied by Kong et al. [56]. 

Mass transfer and local concentration have been studied with the fluorescence method. Hiby experimented on falling films with a pH color indicator exposed to ultraviolet radiation to study the transport and reaction mechanism in the trickle film. This study presented the absorption results as area distribution or images [57]. Schagen and Modigell proposed a fluorescence method for determining the concentration distribution in-depth on thin films at a Reynolds number of 177, studying oxygen absorption in a laminar-wave film containing diacetyl (2,3-butanedione) [58]. Bandi et al. studied oxygen absorption in wavy falling films in two dimensions using a PLIL (planar laser-induced luminescence measurement technique) and a ruthenium complex as an indicator [59]. New measurement methods such as Raman spectroscopy detects local composition in falling films without marker but with the limitation of large measurement times [60].

In addition to measuring film thickness, the near-infrared measurement technique offers the possibility of determining local concentrations of substances in various applications. Matcher et al. used near-infrared spectroscopy to quantify chromophore in tissue based on the absorption spectrum of water [61], and Dorado et al. to determine trace amounts of methanol and glycerol in diesel fuel [62]. Tan et al. determined the sugar content in cherry tomato fruit as an indicator of the quality of the fruit by using near-infrared diffuse reflectance spectroscopy [63]. Dong and Guo studied the apple’s internal quality determining pH, the soluble solids content, and moisture content using near-infrared imaging [64].

Using near-infrared spectroscopy, Pan et al. studied the film thickness, concentration, and temperature of urea dissolved in water. The results of the absorption spectrum at a mass fraction of urea between 0 and 0.40 $g_{urea}g_{total}{}^{-1}$ showed significant absorption differences between 6500 and 7500 ${\mathrm{cm}}^{-1}$. Four lasers and an InGaAs sensor were used in this spectral range as a multiwavelength measurement technique [65]. Kakuta et al. used near-infrared imaging analysis for a temperature measurement combined with concentration determination of ethanol in water during the mixing process in a microchannel. For the study, two halogen lamps with narrow band-pass filters at 1905 and 1935 nm were used with a near-infrared camera with spectral sensitivity between 1000 and 2350 nm [66]. 

Lubnow et al. studied the film thickness and concentration of a urea-water mixture between 0 and 0.40 $g_{urea}g_{total}{}^{-1}$ using a near-infrared measurement technique. The solution was examined from 1250 to 2500 nm using a PbS photodetector. An incandescent lamp is used between 360 and 2600 nm with a filter wheel with band-pass filters at 1350, 1450, 1933, and 2200 nm. The data acquisition for all four wavelengths is almost one minute due to the mechanical function of the filter wheel [67,68].

Medina et al. determined the local concentration and viscosity of glycerol/water mixtures in microchannels with near-infrared imaging using a near-infrared camera and LEDs in this range [69].

The present work provides a non-invasive and simultaneous measurement method to determine the local film thickness distribution and glycerol concentration in falling films under laminar to laminar-wavy flow conditions. An apparatus was developed for the experimental investigations of falling films on a vertical plate. In addition to reaching high viscosities in falling film glycerol was used for the experiments. Glycerol at high concentrations has comparable viscosity to products in evaporation processes. For example, in the sugar industry sucrose solutions are evaporated at temperatures between 90 and 125 °C to reach outlet product concentrations up to 75% (in mass fraction) [2,70]. The sucrose solutions present a dynamic viscosity in this temperature range between 6 and 11 mPa s [1]. Glycerol/water mixtures with glycerol mass fractions between 0 and 0.65 $g_{glyc}g_{total}{}^{-1}$ present a wide dynamic viscosity range up to 15.20 mPa s. Falling films form film thicknesses between 0.5 and 2 mm [71]. Falling films on a vertical plate with Reynolds numbers between 4.8 and 270, and an average film thickness between 0.2 and 1.2 mm were investigated with a high temporal and spatial resolution in two different spectral regions. First, the falling film with fluorescein sodium salt as a fluorescence marker was photographed using a camera in the visible range and an excitation light at 470 nm to calculate the local film thickness. The local glycerol concentrations were determined using a near-infrared camera and high-power LEDs at 1050, 1300, 1450, and 1550 nm in the near-infrared range. The results can support further improvement of the apparatus design and optimization of evaporation processes.

## 2. Materials and Methods

### 2.1. Fluid Preparation

Experiments were carried out at a falling film with a mixture of glycerol-water by using glycerol (ROTIPURAN^®^ ≥ 99.5% p.a., M = 92.09 g/mol, Carl Roth GmbH, Karlsruhe, Germany) and distilled water. Many fats and oils from animals and plants contain glycerol. In these natural fats and oils, glycerol is bound as a triglyceride (fatty acid ester) and occurs as an intermediate product in numerous processes during metabolism [72]. Currently, glycerol is produced from plants by saponification, hydrolytic cleavage, or transesterification methods [72,73,74,75,76,77]. The areas of application for glycerol are wide-ranging. It is an essential ingredient in making skincare products such as creams in cosmetics, often because of its skin compatibility [78]. It is applied as a preservative, flavor, and consistency enhancer in food production for humans and animals [79,80,81,82,83]. It is used as a solvent and moisturizer in the pharmaceutical industry, and in manufacturing, it prevents machinery freezing [84]. It is also involved in producing numerous products, such as paper and textiles. Glycerol is a viscous, colorless, and transparent material with high solubility and high viscosity compared to water. Due to its good solubility in water, 1000 g/L at 25 °C, and the harmlessness of its handling concerning human health, glycerol is very suitable as an experimental liquid. For this purpose, the experiments used solutions with different glycerol mass fractions between 0 and 0.65 $g_{glyc}g_{total}{}^{-1}$. The physical properties of the glycerol-water mixtures at 20 °C are shown in Table 1.

Sucrose solutions are evaporated in falling film evaporators with temperatures between 90 and 128 °C and syrup as the output product with mass concentrations between 72 and 75% are resulted [2]. Here, sucrose syrup presents dynamic viscosities between 8 and 20 mPa s [1], which is similar to glycerol/water-mixtures between 0.50 and 0.65 $g_{glyc}g_{total}{}^{-1}$.

**Figure 1:**
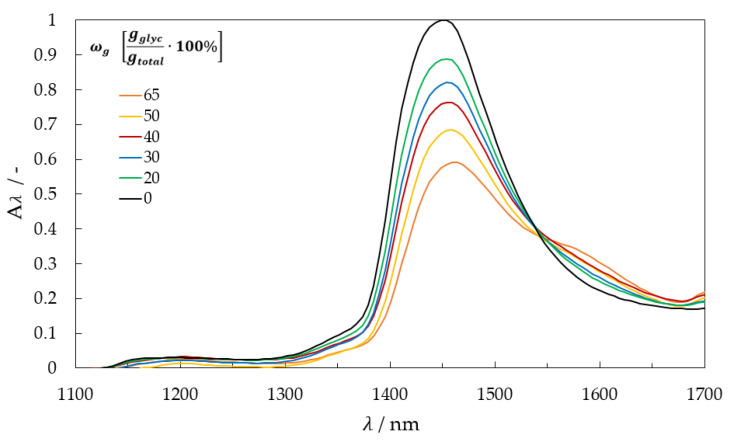
Near-infrared spectra of pure water, glycerol, and glycerol-water mixtures.

The near-infrared absorbance spectra of the experimental fluids were measured using a VIS/NIR spectrometer (light source: CLH600 and NIR module: MCS611, Carl Zeiss Spectroscopy GmbH, Jena, Germany) with an integration time of 5 ms and are shown in Figure 1. The absorbances calculations applied the Beer-Lambert law by using a cuvette with a thickness of 1 mm and an air measurement as a reference.

For experiments on falling films, a wavelength with no absorption is suitable as a reference. For water and glycerol, a reference wavelength of 1050 nm is optimal. The selected high-power LEDs evaluated at difference absorbance were 1300, 1450, and 1550 nm.

### 2.2. Film Thickness Measurement by Using Fluorescence Technique

The fluorescence method is a recognized measurement method for determining the film thickness of fluids. In fluorescence spectroscopy (also known as excitation spectroscopy), the states of molecules are electronically excited by absorption in the visible and ultraviolet spectral regions [89,90]. This spectroscopy technique uses the ability of a molecule at a wavelength $\lambda_{1}$ (where the molecule is excited by externally introduced electromagnetic radiation), to produce spontaneous emission in another wavelength range $\lambda_{2}$, where $\lambda_{2}\;$> $\lambda_{1}$. This spontaneous emission of electromagnetic radiation is called fluorescence. 

Non-invasive optical methods use fluorescent markers with a defined mass concentration to determine film thickness. The intensity of the emitted fluorescence light becomes brighter by increasing film thickness, which means more liquid and thus more fluorescence marker presence. Therefore, the calibration of the film thickness is a function of the change in fluorescence intensity.

**Figure 2:**
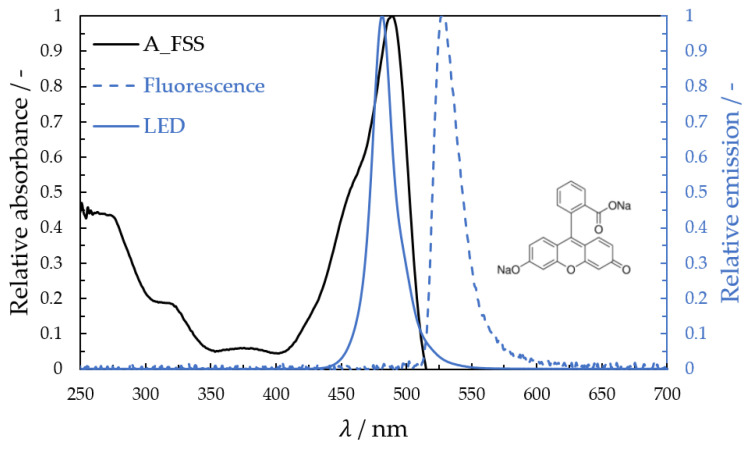
Absorbance and emission spectrum of fluorescein sodium salt and the intensity spectrum of the 470 nm light source.

The fluorescein sodium salt is commonly used due to its absorption and emission in the visible range. Disadvantages of fluorescein are its low fluorescence lifetime, pH sensitivity, and tendency to decompose in the presence of oxygen. The fluorescent indicator fluorescein sodium salt (C_20_H_10_Na_2_O_5_) (FSS, M = 376.27 g/mol, Merck KGaA, Darmstadt, Germany) is a red-orange crystalline powder. It is well soluble in ethyl alcohol, water, and glycerol and is very suitable for many applications and experiments because of its easy handling (no health hazard). The indicator can be excited in both spectral regions, ultraviolet (UV) and visible (VIS). In the UV range, fluorescein has a high absorption between 250 and 350 nm, and in the VIS range, it has a higher absorption peak at about 490 to 492 nm. The emitted spectrum is between 500 and 695 nm, with a maximum emission peak at about 530 nm [91]. Absorption and emission spectra of the dissolved fluorescein sodium salt were measured with a UV/VIS spectrometer (light source: CLD600, sensor module: MCS601, Carl Zeiss Spectroscopy GmbH, Jena, Germany) (see Figure 2). The light source (470 nm) used for the fluorescence measurement is also shown.

Mass fraction of $1.74\cdot 10^{-3}\;g_{FSS}g_{total}{}^{-1}$ of fluorescein sodium salt (FSS) was added to the substance system for film thickness measurements up to 2.4 mm.

### 2.3. Glycerol Mass Concentration Measurement by Using the Near-Infrared Technique

Near-infrared spectroscopy is often implemented in quality control and process monitoring because of the unique information of a substance in this range. The near-infrared spectral range begins at about 750 nm and extends to about 2500 nm. This range contains a combination of physical and chemical information about a substance.

The absorption bands of a molecule in the near-infrared region are defined with the overtones of fundamental vibrations -NH, -OH, and -CH, combination vibrations, and overlap of vibrations. For this reason, near-infrared spectroscopy is based on analyzing groups of frequencies or spectral comparisons of the ratios of the substance of interest to the other components in the analytical sample [92]. The near-infrared spectroscopy technique has the advantage of being non-invasive and in situ without sample preparation. Water has a combination of bands, making analysis more challenging with a significant peak between 1440 and 1460 nm [49].

Transmission and remission are usually used for the near-infrared analysis of a substance. In transmission measurement, the sample is illuminated from one side with a light beam $I_{0}$ [93]. The light is partially absorbed within the sample, leaving the sample with a light intensity I. The fluid layer is illuminated twice in a remission measurement. The incident light $I_{0}$ is diffusely reflected by a background and the reflected light I is received by a sensor [94]. With a smooth background, the difference is that the light is not remitted in all directions, and thus more light arrives at the sensor. However, when the fluid sample rests on a roughened substrate and is struck by a beam of light $I_{0}$ the light is diffusely remitted from the substrate in all directions as it passes through the fluid. For this reason, remission is also called diffuse reflection. 

The partial identification of a substance and quantitative analysis of samples in the near-infrared range is possible by knowing the absorbance curve over the wavelength. The absorbance calculation, which represents the attenuation of light after it has passed through a substance, is carried out using the Beer-Lambert law, see Equation (1). It describes the relationship between the absorbance $A_{\lambda }$ and the concentration *c*, the film thickness *d,* and the molar absorbance coefficient $\varepsilon_{\lambda }$. The absorbance is defined as the decade logarithm of the ratio of irradiated light intensity $I_{0}$ and transmitted light intensity I [95].

(1) $$A_{\lambda }=log_{10}\left(\frac{I_{0}\left(\lambda ,T\right)}{I\;\left(\lambda ,T\right)}\right)=\varepsilon \left(\lambda \right)\cdot c\cdot d$$

### 2.4. Experimental Setup

The sample fluid was circulated during the measurement by a gear pump (MCP-Z with a Z-142 pump head with max. 5.7 L/min, ISMATEC, Wertheim, Germany). The fluid is pumped from the collection tank located under the experimental unit with a capacity of approx. 2 L into the flow tank. The feed tank is filled with small plastic balls to ensure that the fluid level in the collection tank rises as smoothly and without waves as possible [33]. The fluid flowed from a small distributor to the vertical stainless steel plate with flow rates between 0.1 and 1.45 L/min and Reynolds numbers from 2.40 to 463.

**Figure 3:**
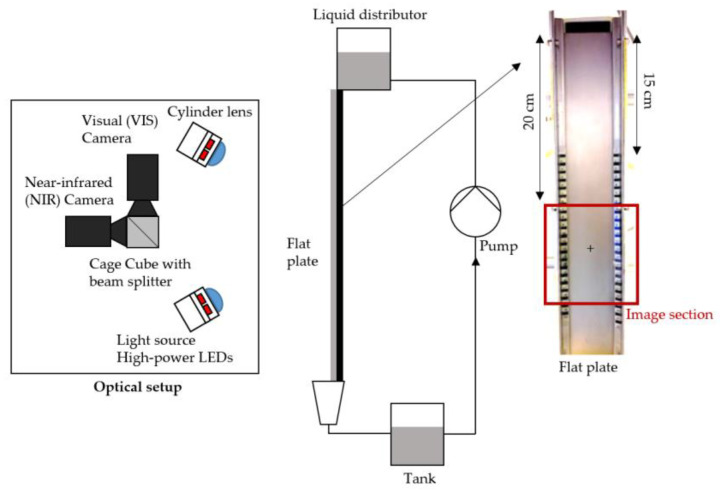
Schematic of the experimental setup for the local investigation of falling films.

The stainless steel plate has a width of 5.2 cm, a length of 40 cm, and is made of steel grade 1.4541. The same material was used for the calibration cuvette. The surfaces of both steel components were treated with a glass bead blasting of grain size 40 to 70 μm, resulting in a rough, diffuse surface that prevents direct reflections. The cameras are placed perpendicular to the falling film plate. The distance between the falling film and the camera is 36 cm, and between the illumination unit and the falling film, it is 17.3 cm. The same distance to the calibration cuvette was configured for the calibration procedure. Markings for the imaging analysis are placed on the edge of the plate, see Figure 3.

Parameters such as the mass flow rate $\dot{m}$, the kinematic viscosity ν, the density ρ, and the plate width of falling film $B_{F}$ determine the Reynolds number, see Equation (2). The pump worked with a volume flow rate between 150 and 1450 mL/min in the experiments.

(2) $$Re=\frac{\Gamma }{\nu }=\frac{\dot{m}}{\nu \rho B_{F}}$$

The Kapitza number is defined as showed in Equation (3) with the density ρ, the surface tension σ, the dynamic viscosity *η*, the gravitational acceleration g and the inclination angle of the falling film plate φ.

(3) $$Ka=\frac{\rho \sigma^{3}}{\eta^{4}g\sin\varphi \;}$$

The average, minimum, maximum, and residual film thickness are defined with the equations in Table 2. with the characteristic length, calculated by Equation (4):

(4) $$\delta_{x}^{+}=\delta_{x}\;{\left(\frac{g\sin\left(\varphi \right)}{\nu^{2}}\right)}^{1/3}$$

**Figure 4:**
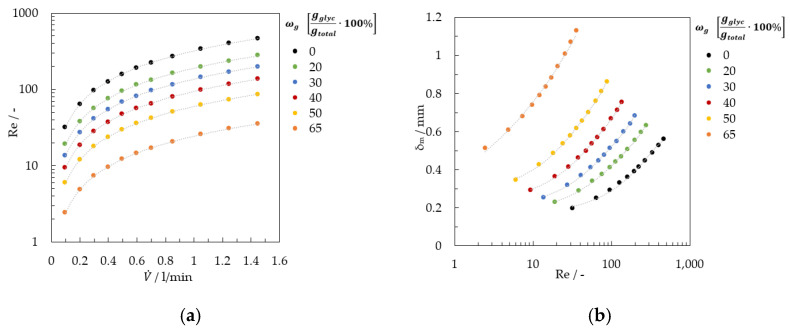
Fluidic characterization of the falling film (**a**) Reynolds number and (**b**) average film thickness.

Figure 4 shows the Reynolds number as a function of volume flow rate and the average film thickness.

**Figure 5:**
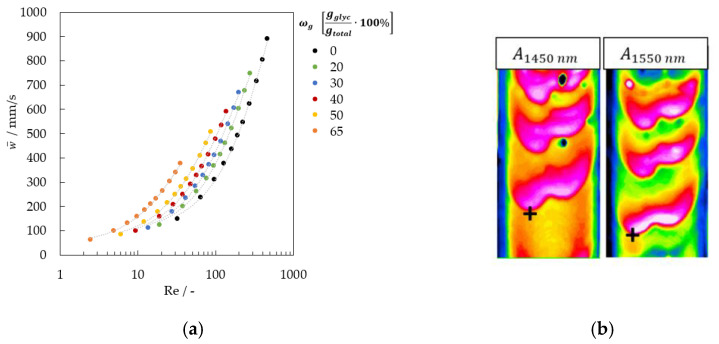
(**a**) Average film velocity and image shift in mm for image acquisition (**b**) example of image shift after 25 ms between 1450 and 1550 nm.

The average film velocity $\bar{w}$ was calculated using Equation (5) and is shown as a function of the Reynolds number in Figure 5.

(5) $$\bar{w}={\left(\frac{\nu \;g\sin\varphi }{3}\right)}^{1/3}Re^{2/3}$$

The average film velocity of the fluid is critical for multiwavelength image analysis. The images with the VIS camera are acquired simultaneously with the NIR images with the sequence: 1050, 1300, 1050, 1450, 1550 nm. The entire image acquisition is completed after 125 ms. This time results from the camera and software integration time for image acquisition and corresponds to a time shift between two images of 25 ms. The change in the position of a fluid element is calculated in mm (Figure 2a).

(6) $$Image_{shift}=\bar{w}\cdot 25\;\mathrm{ms}$$

This image shift is crucial for automatic image processing with multiwavelength analysis. Figure 2b shows the two absorbance images calculated with 1450 and 1550 nm with the reference at 1050 nm and the wave displacement.

### 2.5. Optical Setup

Homogeneous illumination is crucial for imaging analysis efficiency by reducing image processing like filtering or removing noise. However, glossy corrugated and glossy surfaces are difficult for imaging analysis because of reflections. Therefore, selecting a suitable illumination for backgrounds of this type is challenging.

A separate illumination unit with cylinder lenses was designed to generate homogeneous and efficient illumination. The cylinder lenses used are made of optical glass N-BK7 and have a size of 25 mm × 50 mm with a thickness of 11.03 mm and a focal length of 25 mm (Plano-Convex PCX 46-020 uncoated, Edmund Optics, Mainz, Germany). The electronic board with the high-power LEDs is located on the flat surface of the cylinder lens.

High-power LEDs are effective and bright light-emitting diodes that emit light with high power in minimal space. High-power LEDs consume more power than conventional LEDs in a 5 mm epoxy package, which results in more heat generation [96,97,98,99,100,101,102]. Two high-power LEDs at 470 nm (SMB1N-D470, Roithner Lasertechnik GmbH, Vienna, Austria) and four near-infrared LEDs with wavelengths of 1050, 1300, 1450, and 1550 nm (SMB1-1050, SMB1N-1300L, SMB1N-1450, SMB1N-1550S, Roithner Lasertechnik GmbH, Vienna, Austria) were soldered onto the electronic board. The light-emitting diodes have an output power of between 20 and 40 mW.

The image acquisition technique consisted of a near-infrared camera (NIR) and a camera detecting in the visible range (VIS). For NIR image acquisition, the NIR camera with an InGaAs sensor was used (model AVT-Goldeye P-008 SWIR, cooled, Allied Vision Technologies GmbH, Stadtroda, Germany). The operating range of this camera is from 900 to 1700 nm, with a spectral sensitivity between 65 and 70%. The camera’s exposure time is from 5 μs to 1 s with an image acquisition rate of 118 fps (frames per second) and a maximum image resolution of 320 × 256 pixels with a bit depth of 12 bits. The image acquisition in the visual area was performed by the GigE color camera type DFK 33GP1300 with a CMOS Python sensor (The Imaging Source Europe GmbH, 28217 Bremen, Germany). The camera’s resolution is 1280 × 1024 pixels with an image acquisition rate of 90 fps. The image depth of the color images is 24 bits, with 8 bits for each channel red, green, and blue (RGB). The exposure time can be varied from 20 μs to 10 s, and the amplification “gain” is configurable in the system. The evaluation combined a calculation with the camera’s blue (B) and green (G) channels.

The VIS camera is mounted at a 90° angle to the NIR camera and connected with a beam splitter to capture the same image frame and separate the visible and near-infrared regions (T750LPXRXT-UF1 beam splitter, AHF Analysentechnik, Tübingen, Germany). Each camera has integrated lenses: 25 mm focal length for the NIR camera and 16 mm focal length for the VIS camera.

### 2.6. Calibration of Film Thickness and Glycerol Mass Concentration

The calibration was performed by using a calibration cuvette made of stainless steel. The surface was treated with a glass bead blasting and had 24 areas with a depth of 0.1 to 2.4 mm in 0.1 mm steps and two areas with 10 mm. The operating range of falling films is between 0.1 and 2.4 mm. For calibration, the optical setup is aligned with the side of the camera pointing down. The calibration cuvette is filled with the fluid and photographed. 

### 2.7. Imaging Acquisition

Higher flow rates between 0.5 and 1 m/s of the falling film represent a challenge to automated image acquisition. For a slight image shift, the falling film images must be captured very quickly, at a high acquisition rate. Image acquisition with five wavelengths is only possible sequentially for most multiwavelength measurement systems with on/off switching of the LEDs or filter wheels. 

Each absorbance calculation with a wavelength (1300, 1450, or 1550 nm) required a reference image of 1050 nm, so the NIR image series had to be acquired with a short time between each wavelength. For each NIR image acquisition, the 470 nm LEDs were turned on simultaneously. Thus, each NIR measurement is associated with a fluorescence reference for film thickness (VIS image). The VIS and NIR camera acquisition rate affects the total acquisition frame rate. Synchronizing both cameras and the lighting switching for parallel image acquisition proves to be a challenging task. This study resulted in a stable acquisition rate of 40 fps for the integrated image acquisition. The image acquisition sequence is the same for the calibration and the measurement on the falling film.

### 2.8. Imaging Analysis

After acquiring and storing the images, the first step was automatically evaluating the image series for the calibration measurements. Due to the beam splitter, the VIS images were acquired in a mirror image, and NIR images were 90° rotated. After uploading, NIR images were rotated −90°, and VIS images were mirrored. As a result, the two types of images have the exact alignment of the falling film. Then, the ROI (region of interest) for each image type was selected, and the pictures were cropped. VIS images were processed to separate into three color channels red, green, and blue. Then, the blue color channel was subtracted from the green channel and added with a summand of 60. This factor allows the image to be visualized even in the case of a negative result of the subtraction of the channels green minus blue (see Equation (**7**))

(7) $$I\_VIS\_calc=G_{Channel}-B_{Channel}+60$$

The absorbance calculation for three NIR wavelengths is given by Equations (8)–(11).

(8) $$A_{1300}\_calc=log_{10}\left(\frac{I_{1050}}{I_{1300_{\;}}}\right)+K_{1300}$$

(9) $$A_{1450}\_calc=log_{10}\left(\frac{I_{1050}}{I_{1450_{\;}}}\right)+K_{1450}$$

(10) $$A_{1550}\_calc=log_{10}\left(\frac{I_{1050}}{I_{1550_{\;}}}\right)+K_{1550}$$

The ratio of the image reference and NIR image wavelength at 1300, 1450, and 1550 nm is multiplied by 1000. Thus, the intermediate results can be displayed as an image. The result (ratio) calculation also used a factor of 1000 for visualization. The three factors $K_{1300}$, $K_{1450}$ and $K_{1550}$ correct the calibration curves and the absorbance value offset of the falling film images to zero.

### 2.9. Film Thickness Determination by Using Fluorescence Technique

**Figure 6:**
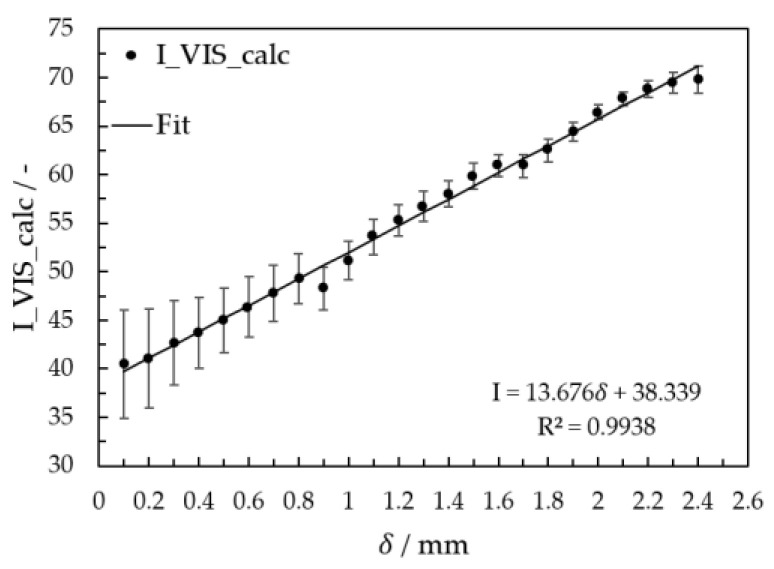
Calibration curves for film thickness calculation with the VIS images.

For calibration of film thickness with the fluorescence method (VIS image acquisition), 36 images were evaluated for each glycerol mass concentration and film thickness. The results of intensity *I* calculated according to Equation (7) are plotted in Figure 6 as a function of film thickness *δ*.

**Figure 7:**
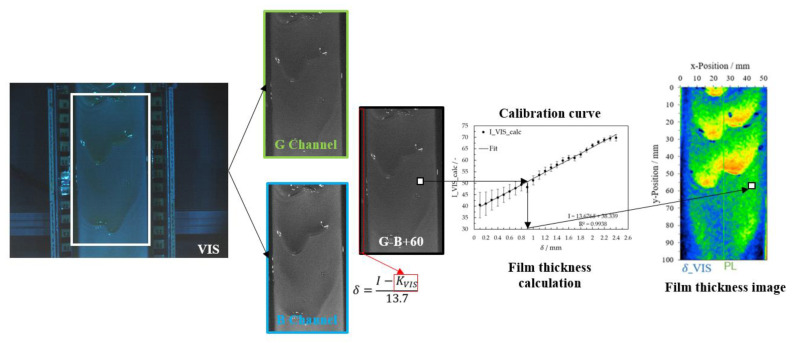
Workflow scheme for determining local film thickness using the VIS image.

Due to the surface tension, solutions have a higher standard deviation at low film thicknesses during the calibration. In the literature, calibration of aqueous solution in the film thickness range of *δ* < 0.6 mm presented this behavior [103]. Additionally, because of the illumination inhomogeneity, the film thickness at 0.9, 1.7, and 2.4 mm deviate from the fitting line. Despite this, the calibration curve fitting has a coefficient of determination of 0.9938 and was used to calculate the film thickness (Equation (11)).

(11) $$\delta =\frac{I-K_{VIS}}{13.7}$$

with $K_{VIS}$ = 38.3. For image analysis on falling films, the correction factor *K* is calculated for each image as the average intensity values at the plate edge and used for calculating the film thickness. The schematic workflow for VIS image processing is shown in Figure 7.

### 2.10. Glycerol Mass Concentration Determination by Using the Near-Infrared Technique

**Figure 8:**
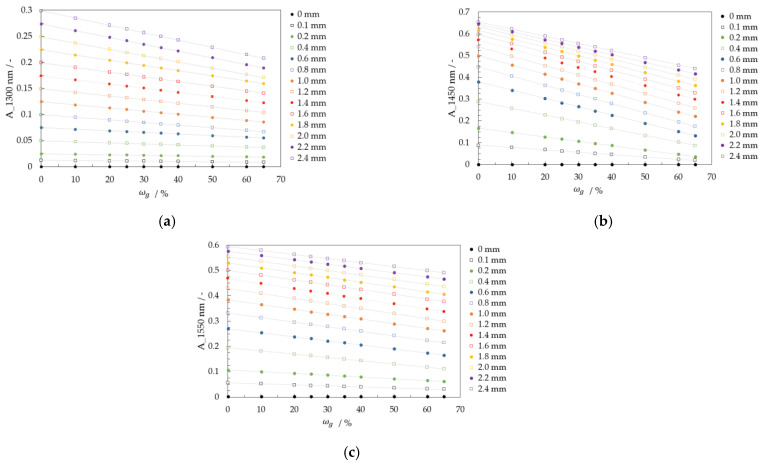
Calibration curves with the NIR technique for the glycerol/water mixture from $\omega_{g}$ = 0 to 0.65 $g_{glyc}g_{total}{}^{-1}$ (**a**) 1300 nm (**b**) 1450 nm and (**c**) 1550 nm.

The absorbances for 1300, 1450, and 1550 nm with the reference are calculated with Equations (8)–(10). Figure 8 displays the calibration curves depending on film thickness δ and glycerol mass concentrations of the samples in $g_{glyc}g_{total}{}^{-1}\cdot$100%.

The calibration curves show the most significant slope for water. For water, the calibration curve at 1300 nm has a maximum absorbance value of 0.3, whereas a mixture with $\omega_{g}$ = 0.65 $g_{glyc}g_{total}{}^{-1}$ has a maximum absorbance of 0.2. The absorbance difference between calibration curves is relevant for the local glycerol mass concentration determination in falling films. For example, the absorbance difference between the calibration curves $\omega_{g}\;$= 0 and 0.10 $g_{glyc}g_{total}{}^{-1}$, and a film thickness of *δ* = 1 mm equals 0.006. Higher film thicknesses have more significant absorbance differences. At 2.4 mm, the absorbance difference between two mixtures is more than twice that for 1 mm, about 0.014. As a result, the evaluation with the wavelength at 1300 nm is suitable for determining glycerol mass concentration at higher film thicknesses. 

To determine the glycerol mass concentration, the calculation with a wavelength at 1450 nm is more suitable than at 1300 nm for low film thickness. Calibration curves, e.g., $\omega_{g}$ = 0 and 0.10 $g_{glyc}g_{total}{}^{-1}$ at *δ* = 1 mm have an absorbance difference of 0.043. This absorbance difference is seven times larger than the calibration curves with 1300 nm (*Δ*A = 0.006).

The evaluation using the 1550 nm wavelength shows a more significant measurement effect than the calibration curves at the 1300 nm wavelength. The absorbance difference between $\omega_{g}\;$= 0 and 0.10 $g_{glyc}g_{total}{}^{-1}$ for a film thickness of *δ* = 1 mm is about 0.019 compared to 0.006 for the calibration curves at the 1300 nm wavelength. On the other hand, the absorbance difference here for the 1550 nm wavelength (*Δ*A = 0.019) is smaller than for the calibration curves at the 1450 nm wavelength (*Δ*A = 0.043). The absorbance difference with the 1450 nm wavelength is more than twice that with the 1550 nm wavelength.

**Figure 9:**
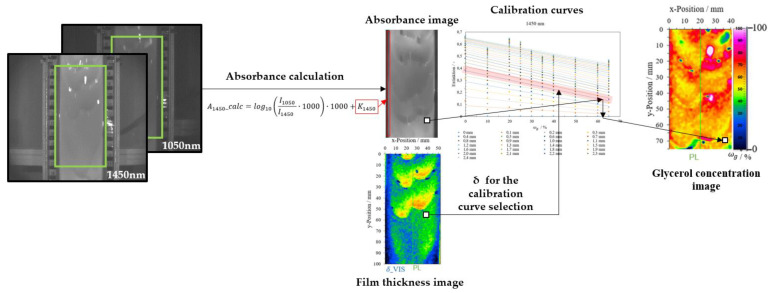
Workflow scheme for the determination of glycerol mass concentration in falling films using NIR image analysis.

The measurement technique allows the determination of local glycerol mass concentrations with a VIS image and only one absorbance image calculated with a NIR wavelength and the reference 1050 nm. For glycerol/water mixtures, the wavelength at 1450 nm is the most suitable because of their absorbance differences. In addition, image analysis uses the information from the film thickness image to determine which calibration curve to use. Finally, the glycerol mass concentration is determined locally using the corresponding calibration curve and the absorbance image. Figure 9 shows the schematic workflow of this image analysis.

The factors *K* are calculated as the average absorbance value of the plate’s border (Equations (8)–(10)). The plate’s surface here is wetted at low film thickness.

### 2.11. Statistical Analysis

Glycerol mass concentration determination $\omega_{g}$ with all three NIR wavelengths was implemented using multiple linear regression. Linear regression is a statistical model to determine the dependence of a variable on one or more independent variables. Multiple linear regression is a variation of this method by using more independent variables. The model in this study is defined by:

(12) $$\omega_{g}=a_{0}+a_{1}\cdot A_{1300}+a_{2}\cdot A_{1450}+a_{3}\cdot A_{1550}$$

The independent variables $A_{\lambda }$ for *λ* = 1300, 1450, or 1550 nm are the absorbance calculated with each near-infrared wavelength and the reference 1050 nm. Based on the calibration, the data of the different glycerol mass concentrations as a function of the film thicknesses were compiled and stored according to the three wavelengths. The absorbance calculations and the glycerol mass concentrations are placed in the matrix. Equation (13) shows the matrix for one film thickness.

(13) $$\left(\begin{array}{c} 0\\ 10\\ 20\\ 30\\ 40\\ 50\\ 60\\ 65 \end{array}\right)\;=\;(\begin{array}{c} 1\;A_{1300}(0)\;A_{1450}(0)\;E_{1550}(0)\\ 1\;A_{1300}(10)\;A_{1450}(10)\;E_{1550}(10)\\ 1\;A_{1300}(20)\;A_{1450}(20)\;E_{1550}(20)\\ 1\;A_{1300}(30)\;A_{1450}(30)\;E_{1550}(30)\\ 1\;A_{1300}(40)\;A_{1450}(40)\;E_{1550}(40)\\ 1\;A_{1300}(50)\;A_{1450}(50)\;E_{1550}(50)\\ 1\;A_{1300}(60)\;A_{1450}(60)\;E_{1550}(60)\\ 1\;A_{1300}(65)\;A_{1450}(65)\;E_{1550}(65) \end{array})\cdot \left(\begin{array}{c} a_{0}\\ a_{1}\\ a_{2}\\ a_{3} \end{array}\right)$$

The regression coefficients $a_{i}$ were determined for each film thickness using Equation (14) and were stored in rows from 0 to 2.4 mm, in steps of 0.1 mm. These will be used to evaluate glycerol mass concentration measurements in falling films.

(14) $$a_{\delta }={\left(A^{\prime }\cdot A\right)}^{-1}\cdot A^{\prime }\cdot \omega_{g}$$

**Figure 10:**
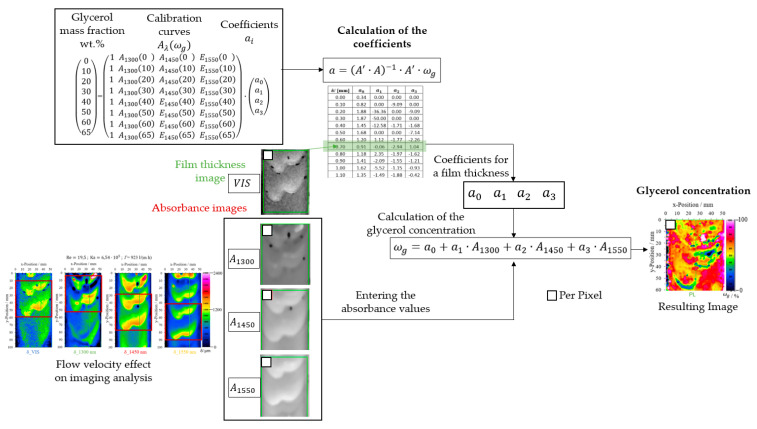
Workflow scheme for determining glycerol mass concentration in falling films using multiwavelength image analysis.

After determining the regression coefficients, the glycerol mass concentration per pixel follows from Equation (12). For this purpose, the corresponding VIS image of the falling film was the input to find the appropriate coefficients in the coefficient matrix for the calculation. Next, a linear interpolation was performed for intermediate film thickness values. After this analysis, an image for the film thickness and an image for the glycerol mass concentration resulted after 125 ms. Figure 10 shows the schematic workflow for the evaluation using multiwavelength image analysis.

In the first step, calibration curves for the wavelengths 1300, 1450, and 1550 nm are read in the software as absorbance values. After determining the coefficients, the glycerol mass concentration is calculated. For the evaluation, the film thickness images calculated with the VIS images were used as input for the linear regression analysis. Specifically, the film thickness images were converted into a matrix, and the measured values were used sequentially to search the coefficients. The exact pixel position is searched and read out in each absorbance image and the calculation was performed according to Equation (12). Then, the result matrix is converted back into an image and displayed in false colors. 

## 3. Results

The fluorescence method for film thickness measurement using the VIS camera was implemented as additional information for the NIR image analysis. For the investigation and validation of the measurement technique, falling films in the laminar to the laminar-wave range were examined.

### 3.1. Results of Film Thickness Determination

**Figure 11:**
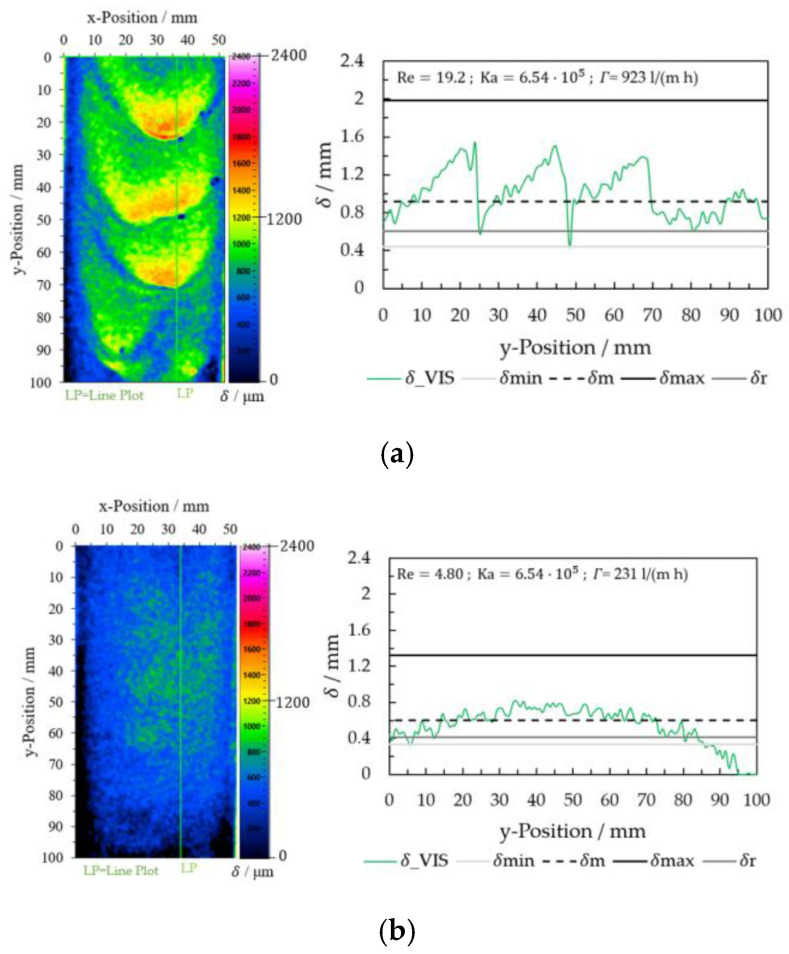
Film thickness determination using VIS image analysis for the glycerol/water mixture 0.65 $g_{glyc}g_{total}{}^{-1}$ (**a**) Re = 19.2 and (**b**) Re = 4.80.

The results were converted into false colors after calculating the film thickness using the VIS images (Equation (11)). The image analysis of different wave characteristics for a glycerol mass concentration of 0.65 $g_{glyc}g_{total}{}^{-1}$ was carried out at varying flow rates. The variation of the film thickness *δ*_VIS for two Reynolds numbers 4.80 (V = 200 mL/min and *Γ* = 231 L/(m h)) and 19.2 (V = 800 mL/min and *Γ* = 923 L/(m h)) is shown in Figure 11 Film thickness values on a line plot at x = 35 mm are displayed on the right side in Figure 11.

**Figure 12:**
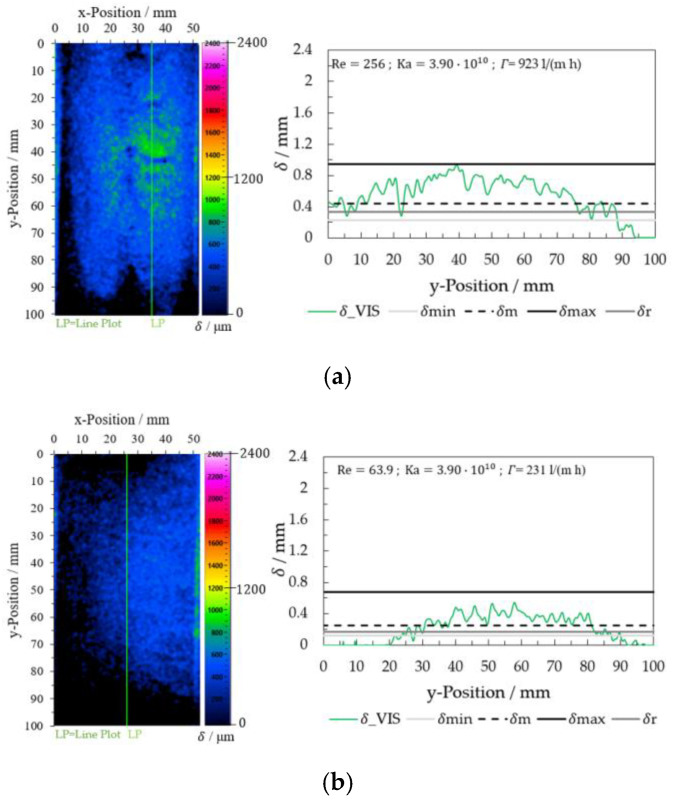
Film thickness determination using VIS image analysis for the glycerol/water mixture 0.65 $g_{glyc}g_{total}{}^{-1}$. (**a**) Re = 19.2 and (**b**) Re = 4.80.

For comparison, Figure 12 shows the results for a mass fraction of glycerol of 0 $g_{glyc}g_{total}{}^{-1}$ for two Reynolds numbers, Re = 256 ($\dot{V}$ = 800 mL/min and *Γ* = 923 L/(m h)) and Re = 63.9 ($\dot{V}$ = 200 mL/min and *Γ* = 231 L/(m h)).

Illumination inhomogeneity affected the evaluation in the two ranges of the plate between y-Position = 0 and 20 mm and y-Position = 80 and 100 mm. Higher film thicknesses (*δ* > 0.8 mm for $\omega_{g}$ = 0.65 $g_{glyc}g_{total}{}^{-1}$ and *Γ* = 923 L/(m h)) show more stable results on the total plate length of 100 mm. The measured film thickness δ_VIS correlated with δmin, δm, and δr. The maximum experimental values on the wave differ from the calculated value δmax by about 20%, which can be attributed to the filtering of the images (image processing). For low film thickness (*δ* < 0.8 mm for $\omega_{g}$ = 0.65 $g_{glyc}g_{total}{}^{-1}$ and *Γ* = 231 L/(m h)) and water (for $\omega_{g}$ = 0 $g_{glyc}g_{total}{}^{-1}$ for *Γ* = 923 L/(m h) and *Γ* = 231 L/(m h)), the results were more sensitive to the illumination quality (inhomogeneities) at the edge of the image (top and bottom). Here, the calculation (I_VIS_cal with Equation (7)) between the G and B channels of the VIS images was insufficient to compensate for the illumination quality.

In the case of water ($\omega_{g}$ = 0 $g_{glyc}g_{total}{}^{-1}$), the falling film had an inhomogeneous distribution of the fluid in the x-direction. Due to the surface tension of the glycerol/water mixtures at low glycerol concentrations ($\omega_{g}$ = 0–0.30 $g_{glyc}g_{total}{}^{-1}$) and the distribution quality of the fluid distributor at the top edge of the plate, the images showed more fluid on the right side than on the left side of the plate. These results can be seen more clearly in Figure 12b.

**Figure 13:**
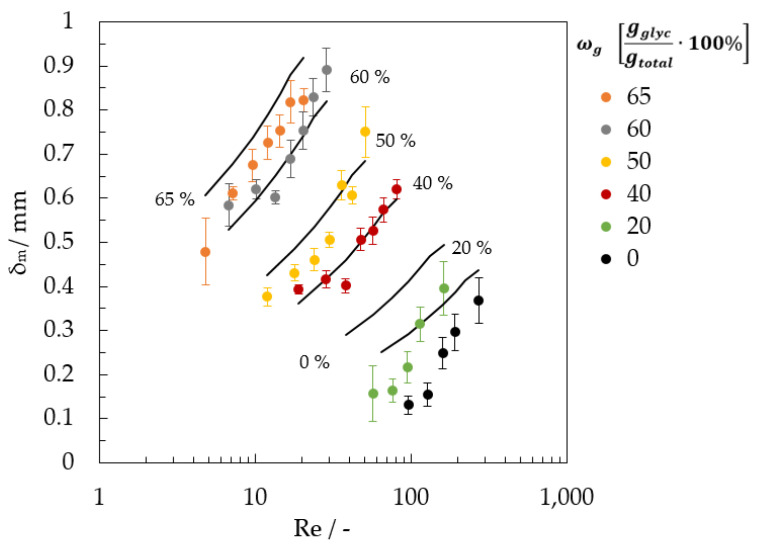
Results of film thickness measurement using VIS image analysis for the falling film with $\omega_{g}$ = 0 (water) and 0.65 $g_{glyc}g_{total}{}^{-1}$.

All measurement series ($\omega_{g}$ = 0 to 0.65 $g_{glyc}g_{total}{}^{-1}$) were averaged arithmetically with 13 images per film thickness. A comparison between the measured mean film thicknesses and the mean film thicknesses [43] is shown in Figure 13. The Reynolds numbers are varied horizontally, and the glycerol mass fractions $\omega_{g}$ vertically.

In measurements with a fixed mass fraction of glycerol and Reynolds numbers variation, the results for low Reynolds numbers (more critical for $\omega_{g}$ < 0.50 $g_{glyc}g_{total}{}^{-1}$) deviated from the calculated film thickness. With a decreased glycerol concentration (between $\omega_{g}$ = 0 and 0.20 $g_{glyc}g_{total}{}^{-1}$), the images have lost information (as black areas), which affects the average calculation. Therefore, this deviation is more significant for low glycerol concentrations, which produce thinner falling film thicknesses. For δ < 0.2 mm there is not sufficient image information for the calculation. The film thickness measurement is suitable for falling film thickness δ > 0.2 mm. In addition, the VIS images show fluctuations in the contour of the film surface. The amplitude of these small fluctuations here is ±0.1 mm. 

### 3.2. Results of Glycerol Concentration Mapping with 1450 nm

**Figure 14:**
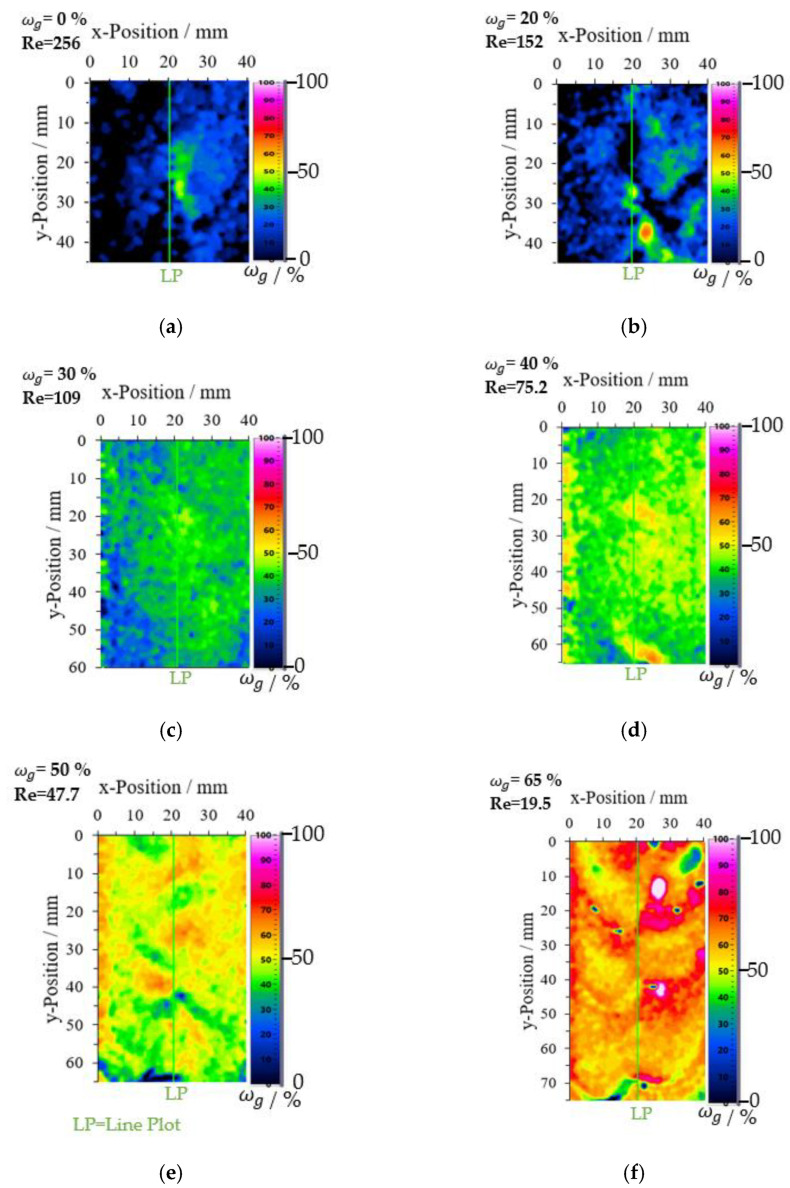
Glycerol mass concentration fields for the falling film from $\omega_{g}$ = 0 to 0.65 $g_{glyc}g_{total}{}^{-1}$ calculated with a film thickness image and the 1450 nm absorbance image. (**a**) $\omega_{g}$ = 0 $g_{glyc}g_{total}{}^{-1}$ and Re = 256 (**b**) $\omega_{g}$ = 0.20 $g_{glyc}g_{total}{}^{-1}$ and Re = 152 (**c**) $\omega_{g}$ = 0.30 $g_{glyc}g_{total}{}^{-1}$ and Re = 109 (**d**) $\omega_{g}$ = 0.40 $g_{glyc}g_{total}{}^{-1}$ and Re = 75.2 (**e**) $\omega_{g}$ = 0.50 $g_{glyc}g_{total}{}^{-1}$ and Re = 47.7 (**f**) $\omega_{g}$ = 0.65 $g_{glyc}g_{total}{}^{-1}$ and Re = 19.5.

A falling film with a fixed glycerol mass fraction was examined to determine the glycerol mass concentration. This method calculated the glycerol mass concentration fields using a 1450 nm absorbance image and a film thickness image (Figure 9). Previous results have shown that at low film thicknesses, suitable measurement points are only available in the center of the image (between y = 20 and 80 mm). For this reason, images with different sizes resulted. Figure 14 shows the resulting concentration fields in false colors for the glycerol/water mixture between $\omega_{g}$ = 0 and 0.65 $g_{glyc}g_{total}{}^{-1}$. For $\omega_{g}$ = 0 $g_{glyc}g_{total}{}^{-1}$, the resulting image has a size of 40 mm × 45 mm, while for $\omega_{g}$ = 0.65 $g_{glyc}g_{total}{}^{-1}$, the resulting image size is 40 mm × 75 mm. 

**Figure 15:**
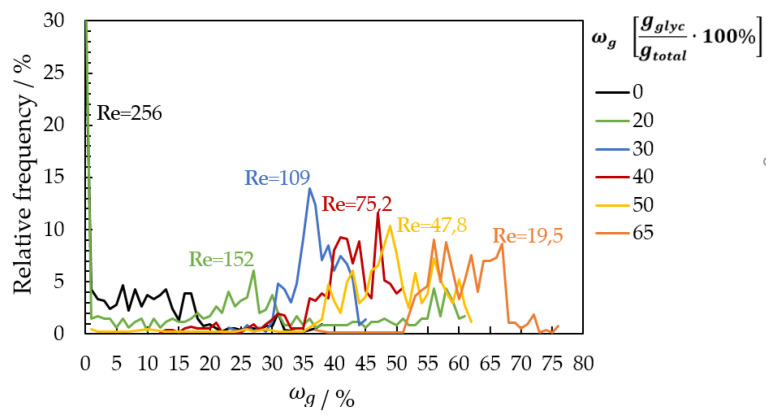
Percent frequency distribution of glycerol mass concentration on a line plot placed in the center of the image.

For a mass fraction of $\omega_{g}$ = 0 $g_{glyc}g_{total}{}^{-1}$ and a Reynolds number of 256, the flow regime is described as “laminar-wave”. The resulting image ($\omega_{g}$ = 0 $g_{glyc}g_{total}{}^{-1}$ in Figure 14a) shows an area in the center of the image, which assumes that a higher glycerol mass concentration is present here. A constant glycerol mass concentration should be present on the entire image. Due to the waveform, the glycerol mass concentration deviates in some image areas. Figure 15 displayed this deviation as percent frequency distribution of glycerol mass concentration. In this measurement ($\omega_{g}$ = 0 $g_{glyc}g_{total}{}^{-1}$), two water strands come out of the liquid distributor and mix at about the center of the plate. These areas show different contours in the waveform in the VIS and NIR images, which deviates the results from the known glycerol mass concentration here. From a mass fraction of $\omega_{g}$ = 0.30 $g_{glyc}g_{total}{}^{-1}$ and a Reynolds number of 109 (flow range = sinusoidal waves), a single fluid strand is formed, allowing uniform concentration fields and correlated to the set glycerol concentration ($\omega_{g}$ = 0.30 $g_{glyc}g_{total}{}^{-1}$ in Figure 14c). 

For $\omega_{g}$ = 0.50 $g_{glyc}g_{total}{}^{-1}$ and a Reynolds number of 47.7, deviations from the expected glycerol mass concentration occur on wave transition areas with $\omega_{g}$ values around 0.40 $g_{glyc}g_{total}{}^{-1}$ ($\omega_{g}$ = 0.50 $g_{glyc}g_{total}{}^{-1}$ in Figure 14e). This deviation becomes more pronounced at $\omega_{g}$ = 0.65 $g_{glyc}g_{total}{}^{-1}$ with Re = 19.5 ($\omega_{g}$ = 0.65 $g_{glyc}g_{total}{}^{-1}$ in Figure 14f). Here, a deviating result in the form of higher or low glycerol mass concentrations resulted due to the contour of the waves, mainly at the wave transition. The 1450 nm wavelength is more sensitive to a slight change in film thickness than the film thickness image calculated with the fluorescence effect and absorbance in the VIS region. A line plot was placed in the center of the image and evaluated for comparison. The glycerol mass concentration fields are displayed in Figure 15 as a relative frequency distribution diagram. The results at the low glycerol mass concentrations $\omega_{g}$ = 0 and 0.20 $g_{glyc}g_{total}{}^{-1}$ deviate strongly from the set glycerol mass concentration. For $\omega_{g}$ > 0.40 $g_{glyc}g_{total}{}^{-1}$, the values approached the set glycerol mass concentration but with broad distribution.

**Figure 16:**
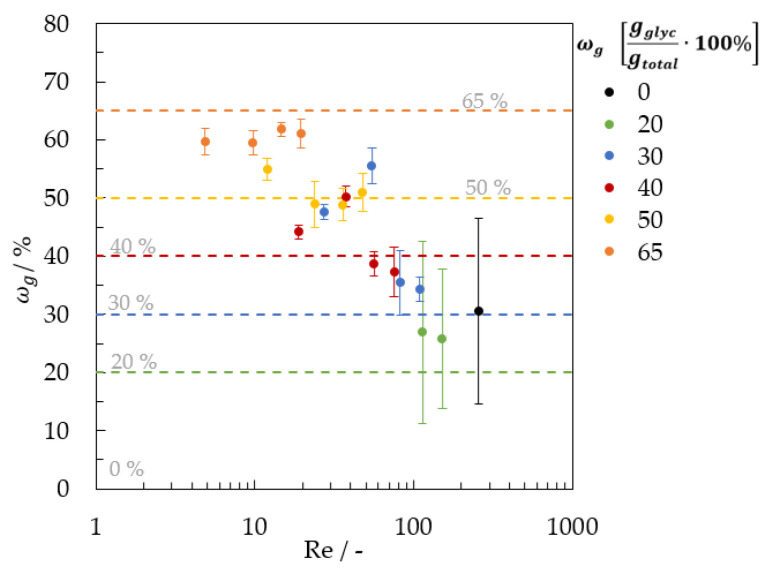
Comparison of measured glycerol mass concentration fields (mean glycerol mass concentration) and set glycerol mass concentration.

The resulting images, including the edges of the falling film, were averaged and compared. The calculation for the different glycerol mass concentrations (from $\omega_{g}$ = 0.30 to 0.65 $g_{glyc}g_{total}{}^{-1}$) were performed for only four Reynolds numbers. For $\omega_{g}$ = 0.20 $g_{glyc}g_{total}{}^{-1}$ two and for $\omega_{g}$ = 0 $g_{glyc}g_{total}{}^{-1}$ one Reynolds number was analyzed. Due to low film thickness, it was not possible to determine the glycerol mass concentration fields on falling films at a low glycerol mass concentration. Figure 16 compares the calculated mean glycerol mass concentration fields, and the set glycerol mass concentration of the investigated falling film.

The results show the highest correlation at higher glycerol mass concentration and Reynolds numbers. For $\omega_{g}$ = 0.65 $g_{glyc}g_{total}{}^{-1}$ and 4.87 ≤ Re ≤ 19.5, all results are similar, with a maximum relative error of about −8.12%. For $\omega_{g}$ = 0.50 $g_{glyc}g_{total}{}^{-1}$, the results show a mean absolute deviation of about 2.19% for Re > 23.9. For $\omega_{g}$ = 0.40 and 0.30 $g_{glyc}g_{total}{}^{-1}$, the measurements deviate strongly at the two smaller Reynolds numbers. For the falling film with a glycerol mass concentration of $\omega_{g}$ = 0.20 $g_{glyc}g_{total}{}^{-1}$, the mean absolute deviation increased to about 34.8%. The relative errors of the measurement are shown in Table 3. A relative error of a maximum of 10% was obtained for glycerol mass concentrations higher as 0.40 $g_{glyc}g_{total}{}^{-1}$ and a minimum film thickness of 0.43 mm.

### 3.3. Results of Glycerol Mass Concentration Mapping with Multiple Linear Regression Analysis

Multiwavelength image analysis combines the synchronization of high-power LEDs in the visible and near-infrared regions with image acquisition and analysis. In addition, multiple linear regression analysis integrated the image analysis with all the wavelengths. 

A crucial parameter for this analysis method is the fluid’s flow velocity and the falling film’s thickness. To perform the analysis with an image section of at least 60 mm in the y-direction, a maximum flow velocity of 254 mm/s and a film thickness of at least 0.4 mm is necessary. This guarantees that the examined fluid element at the beginning of the image series is still visible in the last image. Figure 10 shows an example of the selection of the image section with a size of 50 mm × 50 mm for evaluation with a flow velocity of about 254 mm/s. The position of the fluid element is at the upper edge of the 1300 nm absorbance image and at the lower edge of 1550 nm.

**Figure 17:**
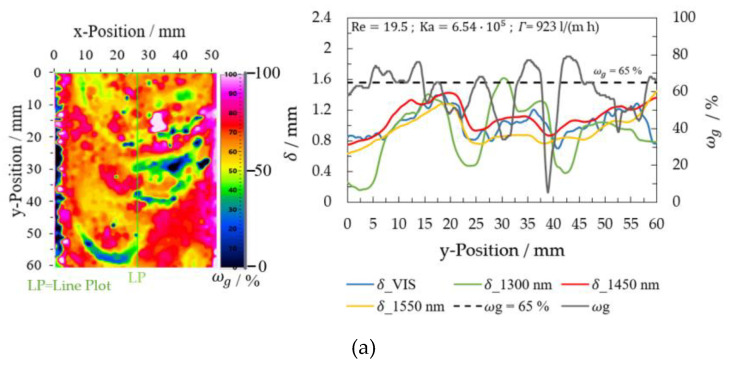

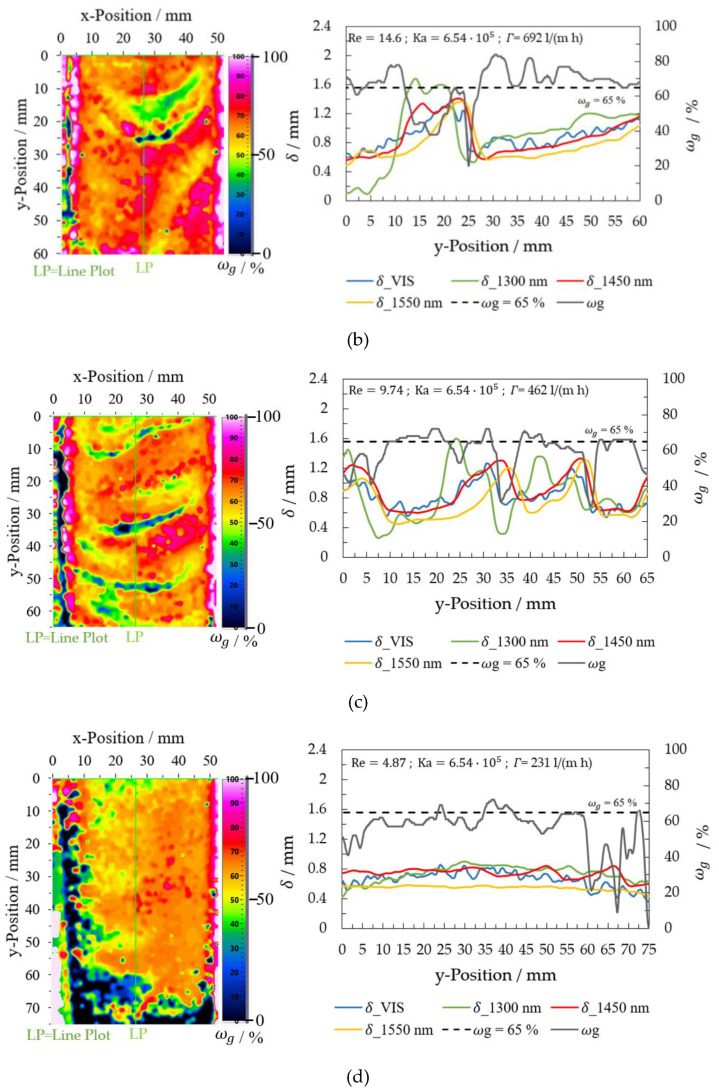
Glycerol concentration fields in falling films at $\omega_{g}$ = 0.65 $g_{glyc}g_{total}{}^{-1}$ calculated using multiwavelength image analysis (**a**) Re = 19.5 (**b**) Re = 14.6 (**c**) Re = 9.74 (**d**) Re = 4.87.

Only the measurement series at $\omega_{g}$ = 0.65 $g_{glyc}g_{total}{}^{-1}$ complies with the two conditions for multiwavelength image analysis presented in this investigation: a low flow rate and a sufficient film thickness. With this glycerol mass concentration, the evaluation and its comparison were performed at different Reynolds numbers. Figure 17 shows in detail the results of the experiments at $\omega_{g}$ = 0.65 $g_{glyc}g_{total}{}^{-1}$ and Reynolds numbers from 4.87 to 19.5. All image measurement points should have a glycerol mass concentration of $\omega_{g}$ = 0.65 $g_{glyc}g_{total}{}^{-1}$ (the set glycerol mass concentration) after the analysis. Due to the differences in the results with the VIS and NIR wavelengths in the waveform, especially at the wave transition in the capillary wave region, the calculated glycerol mass concentration fields show deviations from the set glycerol mass concentration.

For the experiments with a Reynolds number of 19.5, the glycerol mass concentration fields on the line plots show the most significant variations compared to the results at lower Reynolds numbers. For example, the evaluation reaches a glycerol mass concentration of 0.05 $g_{glyc}g_{total}{}^{-1}$ at y-position = 38 mm (Figure 17a). Figure 10 displays this case precisely. Here, the selected section is located at the top edge of the 1300 nm image and at the bottom edge of the 1550 nm image. During the image acquisition, the wave’s shape also changed, influencing the evaluation of wave transition. In addition, a high flow velocity affects the absorbance calculations with NIR wavelengths and the 1050 nm reference incrementing the area with deviations from the set glycerol mass concentration, especially in the wave transition. The 1300 nm evaluation was the most affected due to flow velocity.

As the volume flow rate and the Reynolds number decrease, the fluid flows slower. As a result, imaging acquisition must be performed with a slight shift between frames, making the fluctuations smaller. Figure 17d shows a falling film at *Γ* = 231 L/(m h). The laminar flow range of this mixture is present for Reynolds numbers up to 3.49. In this experiment, the fluid is at the beginning of the “sinusoidal wave” region. Here, the glycerol mass concentration fields are more uniform due to the low amplitude of the waves. At the same time, in thinner film, the images show dry or unwetted areas due to the sensitivity of the measurement method at low film thickness.

## 4. Discussion

Using the fluorescence effect and VIS imaging analysis, film thickness measurements for film thickness *δ* > 0.2 mm were possible, with more stable results between a position of y = 20 and 80 mm. For film thicknesses *δ* < 0.2 mm, the results had areas with no film information, especially in capillary waves and residual film. Moreover, the film surface contour fluctuated due to the sensitivity of the measurement method. Another aspect is the influence of the reference of the 1050 nm wavelength on calculating the absorbances, mainly for the results with the 1300 nm wavelength in the capillary wave region.

The resulting size of the glycerol mass concentration image is determined by the film thickness and the film velocity of the falling film. For film thicknesses *δ* > 0.2 mm, a concentration image with a length of 100 mm is possible. In addition, due to the illumination inhomogeneity at the edge of the 100 mm × 100 mm image section, film thicknesses *δ* < 0.2 mm in the upper and lower edges of the image are not detectable with the fluorescence (VIS calculation), and 1300 nm measurement. Thus, a measurement is only possible in the center of the image with a size of about 80 mm, where information in the form of absorption is still available. 

Flow velocity is a challenge in the image-based measurement of moving fluids. The shift between images during acquisition affects the applicability of the evaluation method. For this reason, a VIS image as a reference for film thickness was acquired for each image taken with NIR wavelength. There is a smaller shift between a NIR image and a VIS image compared to another NIR wavelength, which is improved if the image acquisition synchronizes completely. This slight shift allows evaluation of film thickness with a VIS image and of glycerol mass concentration with a NIR wavelength with less influence on the waveform change over time. The combinations VIS-1300 nm, VIS-1450 nm, and VIS-1550 nm can be used separately in the study of another substance system. In this study, the calibration curves of 1450 nm wavelength for the glycerol/water mixture between 0 and 0.65 $g_{glyc}g_{total}{}^{-1}$ showed a more significant measurement effect. The results had the highest correlation for $\omega_{g}$ > 0.50 $g_{glyc}g_{total}{}^{-1}$ at a broader Reynolds numbers. For falling films at $\omega_{g}$ < 0.50 $g_{glyc}g_{total}{}^{-1}$, the results deviate at lower Reynolds numbers. For $\omega_{g}$ < 0.40 $g_{glyc}g_{total}{}^{-1}$ the best correlation was obtained at Re > 56. Sufficient film thickness and the structure of the waves influenced the evaluation in the area between two fluid strands, where the fluid forms a vortex structure.

Multiwavelength analysis with the multiple regression method used the absorbance images with the three NIR wavelengths and the film thickness image. In this study, a falling film at $\omega_{g}$ = 0.65 $g_{glyc}g_{total}{}^{-1}$ was analyzed with this method. At high glycerol mass concentration, i.e., high viscosity, flow velocity of falling films decreases and film thickness increases. Multiwavelength analysis can be performed more accurately at a maximum flow velocity of 254 mm/s and a film thickness of at least 0.5 mm. In addition, multiwavelength image analysis shows more deviations in the capillary wave region. Due to the temporal change of the film shape and the measurement inaccuracy in the evaluation at 1300 nm, the results show areas with small glycerol mass concentrations, especially in capillary waves (wave transition). Consequently, the glycerol mass concentration differs from the glycerol mass concentration set at the wave transition (for Re = 9.74, 14.6 and 19.5). Here, the results show a low glycerol mass concentration with maximum relative error from 55.4% for Re = 9.74 to 92.3% for Re = 19.5. These results in this area are false information about the glycerol mass concentration, which is hugely influenced by the waveform The best results were obtained with Re = 4.87 with a maximum relative error of 18.5% (10 mm < y-Position < 55 mm) for falling films near to laminar area. In the wave crest region, the results for film thickness *δ* > 1.6 mm had areas with higher glycerol mass concentrations, e.g., $\omega_{g}$ = 1 $g_{glyc}g_{total}{}^{-1}$, attributed to the light reflections and the smoothing filters in image processing. Except for the capillary wave region, the results of the multiwavelength image analysis provide a slighter deviation from set glycerol mass concentrations for a maximum film thickness of 1.2 mm. In addition, Al-Sibai and Schröder showed that efficient heat transfer results in the region of the residual film, which can be followed in more detail experimentally with the measurement method presented here [33,43].

## 5. Conclusions

In the present study, local film thickness and substance concentration, in this case, glycerol in water, in falling films were investigated experimentally using a non-invasive multiwavelength measurement technique. The fluorescence method and near-infrared image analysis were combined to determine these two parameters simultaneously at each location in the falling film. For this purpose, experiments with a glycerol/water mixture in form of a falling film on a vertical plate with glycerol mass fractions between 0 and 0.65 $g_{glyc}g_{total}{}^{-1}$ were carried out, allowing a wide range of film thickness, viscosities, and flow velocities. These operating ranges can be compared to substance systems in evaporation processes such as the evaporation of sucrose solution in the sugar industry. The film thickness determination using the fluorescence method was possible for film thickness δ > 0.2 mm, which enables a direct application on falling film evaporators. For the evaluation, the results of film thickness determination were compared with the calculation of theoretical average film thickness with the best correlation for $\omega_{g}$ > 0.50 $g_{glyc}g_{total}{}^{-1}$ in a wide Reynolds number range. In this range, the dynamic viscosity of the glycerol/water mixture increases from 6 to 15.20 mPa s, which can be compared with the resulting sucrose solution concentration from evaporation processes in the sugar industry.

Evaporation processes have as a result a local change in the substance concentration. Thus, the evaporation performance can be improved by tracking the local substance concentration. In this study, a spatial analysis of the variation of substance concentration was investigated using near-infrared imaging analysis, multi-wavelength lighting, and multiple regression analysis. The experiment results of the multi-wavelength analysis in falling films had the highest correlation at a high mass fraction of glycerol ($\omega_{g}$ = 0.65 $g_{glyc}g_{total}{}^{-1}$) comparing with the set mass concentration of glycerol. Here, the falling films presented low film velocities from 50 to 250 mm/s, demonstrating that the flow velocity leads to image processing limitations and deviations in the results. The film characteristics of the falling film are affected by varying the experimental parameters of volume flow evidencing another limitation of the measurement technique. Depending on the flow characteristics the results deviate mainly in wave transition for laminar-wave flow.

Limiting the analysis to one near-infrared wavelength, the strong influence of flow velocity on the imaging analysis is reduced. For glycerol/water mixtures the wavelength 1450 nm is more suitable due to the absorbance differences in this range. A single-wavelenght analysis reduces the image shift improving the evaluation. The experiments for glycerol mass fraction $\omega_{g}$ > 0.40 $g_{glyc}g_{total}{}^{-1}$ presented the highest correlation in the results. Thus, the single wavelength was more suitable for glycerol/water mixtures in a wide range of mass fractions compared to a multi-wavelength analysis. However, the other wavelengths 1300 and 1550 nm can be implemented for different substance systems involved in evaporation. 

The present study demonstrates the feasibility of determining local film thickness distribution and substance concentrations in falling films using a novel and non-invasive measurement system. Furthermore, the simultaneous experimental determination of the film thickness and the local mass concentration resulting from partial or evaporation can extend the understanding of the influence of these two parameters on mass and heat transport.

## Figures and Tables

**Table 1:** Physical properties of water and glycerol-water mixture at 20 °C [85,86,87,88].

$\omega_{g}\;\left[\frac{g_{glyc}}{g_{total}}\cdot 100\%\right]$	ρ kg m^−3^	η mPa s	n -	Ka -
0	998.2	1.005	1.333	$3.90\times 10^{10}$
10	1019	1.310	1.345	$1.31\times 10^{10}$
20	1041	1.760	1.357	$3.94\times 10^{9}$
30	1065	2.500	1.371	$9.51\times 10^{8}$
40	1091	3.720	1.384	$1.91\times 10^{8}$
50	1117	6.000	1.398	$2.77\times 10^{7}$
60	1143	10.80	1.413	$2.59\times 10^{6}$
65	1155	15.20	1.420	$6.54\times 10^{5}$

**Table 2:** Average, minimum, maximum, and residual non-dimensional film thickness [43].

$\delta_{x}^{+}$	Calculation
$\delta_{min}^{+}$	$1+0.09\;Re^{0.68}$
$\delta_{m}^{+}$	$1+0.615\;Re^{0.47}$
$\delta_{max}^{+}$	$0.67\;Ka^{0.03}\;\left(-3.41+6.24\;Re^{0.19}\right)$
$\delta_{r}^{+}$	$1+0.219\;Re^{0.6}$ $\;für\;Re<25\;Ka^{0.09}$

**Table 3:** Relative errors of the glycerol mass concentration measurement shown in Figure 14 in comparison to the set glycerol mass concentration.

$\omega_{g}\;\left[\frac{g_{glyc}}{g_{total}}\cdot 100\%\right]$	*Γ*/L/(m h)	231	462	692	923
**0**	$\delta_{m}$/mm	0.25	0.33	0.39	0.44
	Relative error/%	-	-	-	+153
**20**	$\delta_{m}$/mm	0.29	0.38	0.44	0.49
	Relative error/%	-	-	+34.8	+29.3
**30**	$\delta_{m}$/mm	0.32	0.41	0.48	0.54
	Relative error/%	+58.4	+85.1	+18.2	+14.4
**40**	$\delta_{m}$/mm	0.36	0.46	0.54	0.60
	Relative error/%	+10.5	+25.6	−3.15	−6.70
**50**	$\delta_{m}$/mm	0.43	0.53	0.62	0.68
	Relative error/%	+10.0	−2.19	−2.20	+1.94
**65**	$\delta_{m}$/mm	0.61	0.74	0.84	0.92
	Relative error/%	−8.12	−8.39	−4.91	−5.97

## Data Availability

Not applicable.
